# Proinflammatory Mediators of Toxic Shock and Their Correlation to Lethality

**DOI:** 10.1155/2010/517594

**Published:** 2010-06-16

**Authors:** Teresa Krakauer, Marilyn J. Buckley, Diana Fisher

**Affiliations:** ^1^Integrated Toxicology Division, U.S. Army Medical Research Institute of Infectious Diseases, Fort Detrick, MD 21702-5011, USA; ^2^Statistics Division, U.S. Army Medical Research Institute of Infectious Diseases, Fort Detrick, MD 21702-5011, USA

## Abstract

Bacterial exotoxins and endotoxins both stimulate proinflammatory mediators but the contribution of each individual toxin in the release of mediators causing lethal shock is incompletely understood. This study examines the cytokine response and lethality of mice exposed to varying doses of staphylococcal enterotoxin B (SEB) or lipopolysaccharide (LPS) and their combinations. In vivo, SEB alone induced moderate levels of IL-2 and MCP-1 and all mice survived even with a high dose of SEB (100 *μ*g/mouse). LPS (80 *μ*g/mouse) caused 48% lethality and induced high levels of IL-6 and MCP-1. SEB induced low levels of TNF*α*, IL-1, IFN*γ*, MIP-2, and LPS synergized with SEB in the expression of these cytokines and that of IL-6 and MCP-1. Importantly, the synergistic action of SEB and LPS resulted in lethal shock and hypothermia. ANOVA of cytokine levels by survival status of SEB-plus-LPS groups revealed significantly higher levels of TNF*α*, IL-6, MIP-2, and MCP-1 in nonsurvivors measured at 8 hours. Significantly higher levels of IFN*γ* and IL-2 were observed at 21 hours in nonsurvivors of toxic shock compared to those in survivors. Overall, synergistic action of SEB and LPS resulted in higher and prolonged levels of these key cytokines leading to toxic shock.

## 1. Introduction

Bacterial exotoxins and endotoxins are among the most common etiological agents that cause septic shock [1–3]. Although similar cytokines are released from host cells stimulated with these structurally distinct bacterial products, the stimulants act through distinct cell surface receptors on host cells. Staphylococcal enterotoxin B (SEB) and structurally related exotoxins are bacterial superantigens that potently activate antigen-presenting cells by binding directly to major histocompatibility complex (MHC) class II molecules [1, 4, 5]. These exotoxins also interact with specific V*β* regions of the T cell antigen receptors resulting in polyclonal T cell activation [6]. Interactions of superantigens with antigen-presenting cells and T cells lead to massive proinflammatory cytokine and chemokine release, causing clinical symptoms that include fever, hypotension, and shock [1, 2, 4, 7, 8]. 

 In contrast, lipopolysaccharide (LPS) from gram-negative bacteria binds to a different receptor on monocytes/macrophages. An LPS-binding protein in serum first binds to LPS and facilitates its binding to cell surface protein CD14 on monocytes/macrophages and other cells [9, 10]. The subsequent interaction of LPS/CD14 complex with Toll-like receptor 4 on these cells initiates recruitment of intracellular adaptors and downstream signaling pathways activating NF*κ*B and results in hyperproduction of proinflammatory cytokines and chemokines [10, 11]. High levels of these mediators induce systemic inflammatory response, vascular collapse, and shock [12].

 In humans, either SEB or LPS alone can induce shock as humans are very sensitive to these bacterial products [2, 12, 13]. Current in vivo investigations on SEB-induced pathogenesis have relied heavily on murine models of lethal shock. However, mice are less susceptible (compared to humans) to SEB due to the decreased affinity of SEB to mouse MHC class II molecules [14, 15] and enhancing agents are used in addition to SEB [16–21]. Potentiating agents such as LPS, D-galactosamine, or actinomycin D were used to amplify the toxic effects of SEB in vivo [18–21]. In these various animal models, there is a strong correlation between toxicity and increased serum levels of inflammatory mediators, TNF*α*, IL-1, IL-6, and IFN*γ* [16–19]. However, the contribution of each individual cytokine or chemokine to toxicity has not been completely delineated. This study was undertaken to investigate the effects of different doses of SEB, LPS, and SEB plus LPS on the cytokine response and survival of mice exposed to these bacterial products.

## 2. Materials and Methods

### 2.1. Reagents

SEB was obtained from Toxin Technology (Sarasota, FL). The endotoxin content of these SEB preparations was <1 ng of endotoxin per mg of SEB as determined by the Limulus amoebocyte lysate gelation test (Biowhittaker, Frederick, MD). *Escherichia coli* 055:B5 LPS was purchased from Difco Laboratories (Detroit, MI) and reconstituted in sterile PBS. Frozen (−70°C) aliquots of SEB and LPS were used for all subsequent studies. 

### 2.2. Mouse Model of Lethal Shock

Male Balb/c mice, weighing ~20 g each (7–10 weeks old), were obtained from NCI (Frederick, MD). Mice were housed in conventional microisolator cages with food and water freely available at all times. SEB and LPS were administered intraperitoneally (i.p.; 200 *μ*L) with a tuberculin syringe (26 G–3/8 inch needle). When SEB and LPS were used together, LPS was injected 4 hours after SEB as this was the optimal time previously determined to cause septic shock [19, 22–24]. All injections (0.2 mL/mouse) were given i.p. and all dilutions were made in saline. Doses of SEB used in survival experiments range from 1 to 100 *μ*g/mouse as described in figure legends. A single high dose of LPS (80 *μ*g/mouse) was used (*n* = 27). The effect of SEB alone, either 30 *μ*g/mouse (*n* = 20) or 100 *μ*g/mouse (*n* = 10) was also examined. Survival experiments were conducted with the different doses of SEB alone, LPS alone, and varying doses of SEB and LPS in groups of 10 to 15 mice per dose. Experiments were repeated multiple times as indicated in figures. The following combinations of SEB plus LPS were used: SEB 1 *μ*g/mouse plus LPS 60 *μ*g/mouse (*n* = 20), SEB 1 *μ*g/mouse plus LPS 80 *μ*g/mouse (*n* = 63), SEB 10 *μ*g/mouse plus LPS 10 *μ*g/mouse (*n* = 30), SEB 30 *μ*g/mouse plus LPS 10 *μ*g/mouse (*n* = 40), SEB 60 *μ*g/mouse plus LPS 10 *μ*g/mouse (*n* = 40), SEB 100 *μ*g/mouse plus LPS 10 *μ*g/mouse (*n* = 10), SEB 30 *μ*g/mouse plus LPS 1 *μ*g/mouse (*n* = 25), and SEB 100 *μ*g/mouse plus LPS 1 *μ*g/mouse (*n* = 10). Mice exposed to both SEB and LPS or high dose of LPS alone succumbed to death between 20 to 141 hours with the majority (97%) of death occurring between 21 and 70 hours after initial toxin dose. Lethal end points were monitored twice per day for 2 weeks. Temperature-based experiments were performed with each animal subcutaneously implanted (6–10 days before toxin exposure) with a Biomedic Data Systems (Seaford, DE) transponder located on the dorsum between the shoulder blades [24].

 Animal research was conducted in compliance with the Animal Welfare Act as well as other federal statutes and regulations. The facility where this research was conducted is fully accredited by the Association for Assessment and Accreditation of Laboratory Animal Care International. All efforts adhered to principles stated in the Guide for Care and Use of Laboratory Animals (National Research Council). 

### 2.3. Cytokine and Chemokine Assays

Previous results indicated that the optimal timing for serum cytokine collection after mice were given toxins is 8 hours after SEB [19, 22, 23]. Serum from an individual mouse was collected at 8 hours and 21 hours after SEB, from anesthetized mice retro-orbitally or by cardiac puncture. Most experimental groups (*n* = 10) were repeated multiple times over a 1.5-year period. Sera were stored at −70°C until assay in duplicates by ELISA. TNF*α*, IL-1, IL-2, IL-6, IFN*γ*, MIP-2, and MCP-1 were measured by ELISA using cytokine-specific antibodies and cytokine standards from R&D Systems (Minneapolis, MN) according to instructions of the manufacturers [19, 22, 23]. A standard curve was generated for each plate using five dilutions of cytokine standards. The detection limit for cytokine ELISA was 10 pg/mL. Background levels of each cytokine/chemokine, all found to be negligible, were derived from a prebleed of the same mice performed 2–4 days before each experiment.

### 2.4. Statistical Analysis

Data were analyzed with the use of SAS software, version 9.2 (Cary, NC). Differences in cytokine/chemokine levels between treatment groups were assessed by obtaining geometric means with 95% confidence intervals. Statistical comparisons of survival and cytokine/chemokine data were performed using two-tailed Fisher's exact tests and analysis of variance (ANOVA), respectively. All reported *P-*values are two sided, and a *P* value of less than .05 was considered to indicate statistical significance.

## 3. Results

### 3.1. Survival Analysis of Varying Doses of SEB, LPS, and SEB in Combination with LPS

**Figure 1:**
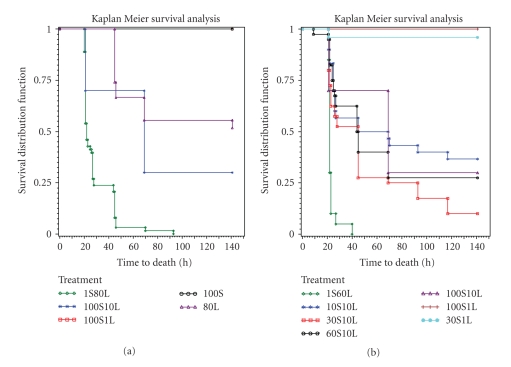
Survival analysis of BALB/c mice treated with (a) 1 *μ*g SEB plus 80 *μ*g LPS (*n* = 63), 100 *μ*g SEB plus 10 *μ*g LPS (*n* = 10), 100 *μ*g SEB plus 1 *μ*g LPS (*n* = 10), 100 *μ*g SEB (*n* = 10), and 80 *μ*g LPS (*n* = 27). (b) 1 *μ*g SEB plus 60 *μ*g LPS (*n* = 20), 10 *μ*g SEB plus 10 *μ*g LPS (*n* = 30), 30 *μ*g SEB plus 10 *μ*g LPS (*n* = 40), 60 *μ*g SEB plus 10 *μ*g LPS (*n* = 40), 100 *μ*g SEB plus 10 *μ*g LPS (*n* = 10), 30 *μ*g SEB plus 1 *μ*g LPS (*n* = 25), and 100 *μ*g SEB plus 1 *μ*g LPS (*n* = 10).

Mice exposed to 30 *μ*g (*n* = 20) or 100 *μ*g (*n* = 10) of SEB all survived. Exposure to 80 *μ*g LPS alone (*n* = 27) resulted in 52% survival, significantly different from mice treated with SEB alone (*P* < .05) (Figure 1(a)). Survival rates of mice exposed to 30 *μ*g SEB plus 1 *μ*g LPS (*n* = 25) or 100 *μ*g SEB plus 1 *μ*g LPS (*n* = 10) were 96% and 100%, respectively, and did not differ significantly from mice singularly treated with SEB (Figure 1(b)). Increasing LPS 10 fold to 10 *μ*g/mouse with varying doses of SEB yielded a significant difference in survival rates from mice exposed to SEB alone (*P* < .05). Mice exposed to 10 *μ*g SEB (*n* = 30) or 30 *μ*g SEB (*n* = 40), 60 *μ*g SEB (*n* = 40), or 100 *μ*g (*n* = 10) with a constant amount of LPS (10 *μ*g) had 37%, 10%, 27%, and 30% survival rates, respectively (Figure 1(b)). There were no survivors when mice were exposed to 1 *μ*g/mouse SEB and 80 *μ*g/mouse LPS (*n* = 63) or 1 *μ*g/mouse SEB and 60 *μ*g/mouse LPS (*n* = 20) (Figure 1). Increasing SEB dose with a relatively low amount of LPS (10 *μ*g) did not affect survival rates significantly whereas higher LPS doses (either 60 or 80 *μ*g) plus a low dose of SEB (1 *μ*g) caused complete mortality. It appears that systemic shock using SEB plus LPS combinations in this mouse model was driven to a large extent by LPS although synergism with SEB was necessary. 

### 3.2. Hypothermia in Nonsurvivors of Toxic Shock

**Figure 2:**
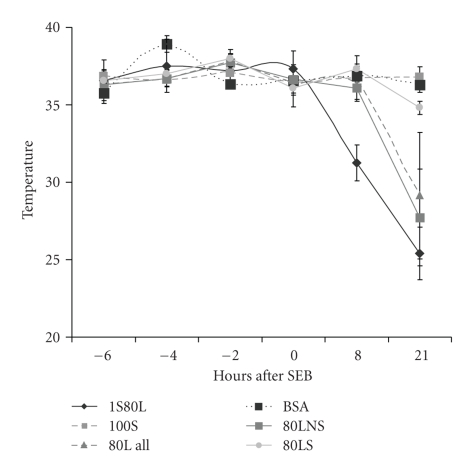
Temperature changes of BALB/c mice treated with 100 *μ*g SEB, 80 *μ*g LPS, 1 *μ*g SEB plus 80 *μ*g LPS, or 100 *μ*g of bovine serum albumin (BSA). Points represent the mean ± standard deviation (SD) for each group (*n* = 10) except for survivors (80LS) and nonsurvivors (80LNS) of mice treated with 80 *μ*g LPS.

In addition to lethality as an endpoint, body temperature was also used as a marker of systemic shock as previous studies showed hypothermia precedes death in SEB-induced shock [24]. Figure 2 shows that mice given SEB alone maintained normal body temperature similar to temperature of control mice exposed to bovine serum albumin. However, mice exposed to 1 *μ*g/mouse SEB and 80 *μ*g/mouse LPS experienced hypothermia as early as 8 hours after SEB and temperature continued to drop dramatically. Interestingly, mice exposed to LPS alone (80 *μ*g/mouse) had a slight temperature drop of 2°C at 8 hours and moderate hypothermia was recorded at 21 hours. As 50% of mice survived in this group, re-examination of temperatures of nonsurvivors and survivors in the LPS-treated group separately revealed that hypothermia was experienced only in nonsurvivors (Figure 2). Thus, there is a good correlation between lethality and hypothermia regardless of the toxin and body temperature is an accurate indicator of systemic shock. 

### 3.3. Effect of SEB, LPS, and Varying Doses of SEB Plus LPS on Serum Cytokines and Chemokines

Proinflammatory mediators have critical pathophysiologic effects in vivo and many of the manifestations of septic shock have been correlated to the exaggerated release of these cytokines upon interaction of host cells with SEB and/or LPS [1, 2, 7, 8, 12]. We and others showed that serum TNF*α*, IL-1, IL-6, and IFN*γ* were critical in inducing lethality in murine models of SEB-induced shock [16, 19, 23]. Here we also examined the serum levels of these cytokines and two chemokines, MIP-2 and MCP-1, from mice exposed to varying doses of SEB plus LPS as all mice survived, even at 100 *μ*g of SEB. Two time points, 8 and 21 hours were chosen as some cytokines such as TNF*α* and IL-1 are induced relatively early [19] and the later time at 21 hours might reveal the cytokines induced later by syngergistic effects. The 21 hours time point is also the time near the lethal end point. 

**Figure 3:**
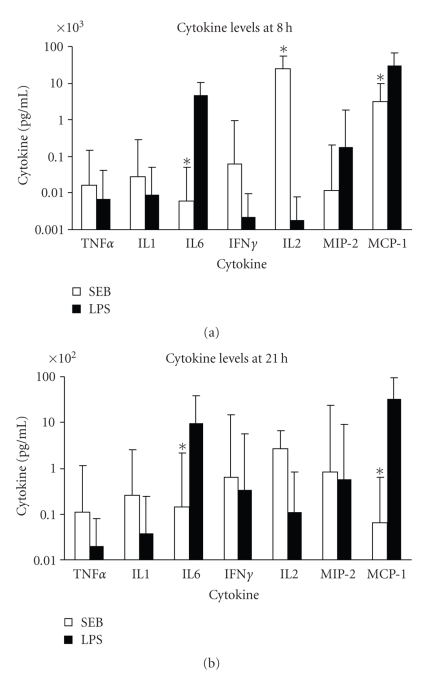
Serum levels of TNF*α*, IL-1, IL-6, IFN*γ*, IL-2, MIP-2, and MCP-1 at (a) 8 hours after SEB or LPS administration (b) 21 hours after SEB or LPS administration. Points represent the geometric mean ± standard deviation (SD) for each group. SEB group consisted of mice treated with 100 *μ*g SEB and LPS group was treated with 80 *μ*g LPS. The “*” indicates *P *< .05 between groups treated with SEB or LPS.

 We first investigated serum cytokines levels of mice treated with either SEB or LPS alone. SEB (100 *μ*g/mouse, *n* = 10) induced high levels of IL-2 and MCP-1, 24174 pg/mL and 3103 pg/mL, respectively, measured at 8 hours after SEB (Figure 3(a)). Both of these cytokines dropped dramatically to 272 pg/mL and 6 pg/mL for IL-2 and MCP-1, respectively at 21 hours. In contrast, LPS (80 *μ*g/mouse, *n* = 16) induced significantly higher levels of IL-6, MIP-2, and MCP-1 (4584 pg/mL, 179 pg/mL, 29721 pg/mL) but negligible IL-2 (2 pg/mL) when compared to the SEB-treated group at 8 hours. This is not surprising as IL-2 is a T cell growth factor mostly induced by T-cell mitogens or stimulants. At this early time point, a 6000-fold higher IL-6 and 10-fold higher MCP-1 were seen in the LPS-treated group when compared to the SEB-treated group. The low level of TNF*α* induced by LPS seen here was likely due to the sampling time after LPS exposure (4 hours) as TNF*α* peaked 90 minutes to 120 minutes after LPS and disappeared within 6 to 8 hours [24, 25]. The sampling time of 8 hours after SEB (4 hours after LPS) was a compromise time chosen to accommodate the other cytokines/chemokines that appeared later after TNF*α*. At 21 hours after toxin exposure, all cytokines decreased to lower levels (>100 pg/mL) except that of IL-6 (983 pg/mL) and MCP-1 (3341 pg/mL) in LPS-treated mice and 272 pg/mL of IL-2 in SEB-treated mice (Figure 3(b)). Pairwise comparison of mice treated singularly with SEB or LPS indicated the levels of IL-6, IL-2, and MCP-1 were significantly different between the two groups at 8 hours and IL-6 and MCP-1 remained significantly different at 21 hours.

**Figure 4:**
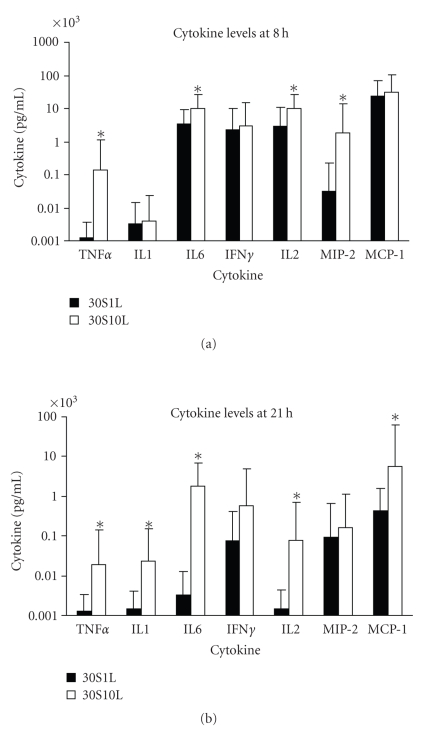
Serum levels of TNF*α*, IL-1, IL-6, IFN*γ*, IL-2, MIP-2, and MCP-1 of mice treated with 30 *μ*g SEB + 1 *μ*g LPS or 30 *μ*g SEB + 10 *μ*g LPS (a) 8 hours after SEB administration (b) 21 hours after SEB administration. Values represent the geometric mean ± SD for each group. The “*” indicates *P *< .05 when comparing the two groups.

 We also analyzed and compared the cytokine levels between two groups of mice treated with a constant SEB dose of 30 *μ*g but different low LPS doses of 1 *μ*g (*n* = 24) or 10 *μ*g (*n* = 14). Figure 4(a) shows significantly higher TNF*α*, IL-6, IL-2, and MIP-2 were found in the 30 *μ*g SEB plus 10 *μ*g LPS group at 8 hours. At 21 hours, significantly higher TNF*α*, IL-1, IL-6, IL-2, and MCP-1 were found in the 30 *μ*g SEB plus 10 *μ*g LPS group compared to the 30 *μ*g SEB plus 1 *μ*g LPS group (Figure 4(b)). Thus there was a substantial potentiation of cytokines and chemokines with the higher LPS dose when SEB was kept constant. Pairwise comparison of these two groups showed prolonged higher levels of TNF*α*, IL-6, and MCP-1 levels in the higher LPS dose (10 *μ*g) group. It appears that the higher cytokine response, especially at the later time point, was also influenced mostly by the LPS dose. 

**Figure 5:**
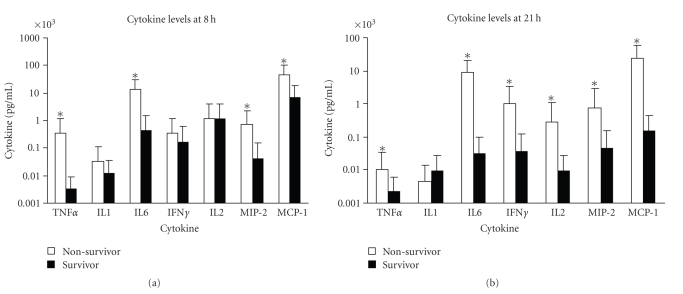
Serum levels of TNF*α*, IL-1, IL-6, IFN*γ*, IL-2, MIP-2, and MCP-1 of mice treated with SEB + LPS (a) 8 hours after SEB administration (b) 21 hours after SEB administration by survival status. Points represent the geometric mean ± standard deviation (SD) for survivors and nonsurvivors of mice treated with SEB + LPS. SEB + LPS group consisted of mice treated with 1 *μ*g SEB + 80 *μ*g LPS, 1 *μ*g SEB + 60 *μ*g LPS, 10 *μ*g SEB + 10 *μ*g LPS, 30 *μ*g SEB + 10 *μ*g LPS, 60 *μ*g SEB + 10 *μ*g LPS, 100 *μ*g SEB + 10 *μ*g LPS, 30 *μ*g SEB + 1 *μ*g LPS, and 100 *μ*g SEB + 1 *μ*g LPS. Values represent the geometric mean ± SD for each group. The “*” indicates *P *< .05 when survivors were compared with nonsurvivors of toxic shock.

 The cytokine response using a combination of varying doses of SEB with LPS, SEB 30 *μ*g/mouse plus LPS 1 *μ*g/mouse (*n* = 24), SEB 100 *μ*g/mouse plus LPS 1 *μ*g/mouse (*n* = 10), SEB 10 *μ*g/mouse plus LPS 10 *μ*g/mouse (*n* = 5), SEB 30 *μ*g/mouse plus LPS 10 *μ*g/mouse (*n* = 14), SEB 60 *μ*g/mouse plus LPS 10 *μ*g/mouse (*n* = 10), SEB 100 *μ*g/mouse plus LPS 10 *μ*g/mouse (*n* = 3), SEB 1 *μ*g/mouse plus LPS 60 *μ*g/mouse (*n* = 10), and SEB 1 *μ*g/mouse plus LPS 80 *μ*g/mouse (*n* = 34), was also analyzed. As shown in Figure 1, most mice survived the lower dose of LPS (1 *μ*g/mouse) plus SEB whereas survival decreased substantially with the higher LPS dose (10 *μ*g/mouse) plus various SEB doses. ANOVA was used to analyze the various cytokine and chemokine levels between survivors and nonsurvivors of treatment groups exposed to both SEB and LPS. In mice treated with both SEB and LPS, significant differences (*P* < .05) in levels of TNF*α* (2 pg/mL in survivors versus 535 pg/mL in nonsurvivors), IL-6 (2531 pg/mL in survivors versus 15325 pg/mL in nonsurvivors), MIP-2 (31 pg/mL in survivors versus 945 pg/mL in nonsurvivors), and MCP-1 (9672 pg/mL in survivors versus 50142 pg/mL in nonsurvivors) measured at 8 hours after SEB administration (Figure 5(a)). In addition to these cytokines/chemokines, significant higher levels of IFN*γ* (84 pg/mL in survivors versus 1262 pg/mL in nonsurvivors) and IL-2 (8 pg/mL in survivors versus 385 pg/mL in nonsurvivors) were observed 21 hours after SEB administration (Figure 5(b)). More importantly, IL-6, MIP-2, and MCP-1 levels remained high at the later time point in nonsurvivors. Overall, this analysis of cytokine levels by survival status agreed with the data from nonsurvivors and survivors of all groups of mice treated singularly with SEB, LPS, or both SEB and LPS.

## 4. Discussion

The exaggerated systemic response to SEB is similar to LPS where excessive proinflammatory cytokine release causes increase in vascular permeability, cell adhesion, and coagulation [2, 12]. Either SEB or LPS alone induce these proinflammatory mediators and it is not clear whether there is a threshold level of these mediators above which either singularly or in combination they can trigger shock. LPS naturally synergizes with superantigens to induce the proinflammatory cascade culminating in shock. Moreover, the cytokines, TNF*α*, IL-1, and IFN*γ* induced by either SEB or LPS also synergizes with each other to promote inflammation [26]. In this study we investigated the contribution of the cytokines, TNF*α*, IL-1, IL-6, IFN*γ*, and IL-2, and chemokines, MIP-2 and MCP-1, to lethal shock in mice exposed to SEB or LPS and their combination. Our survival analysis indicated that increasing LPS dose had more effect in influencing mortality than SEB dose with SEB plus LPS-induced shock. The cytokine data also showed that a higher LPS dose with a constant SEB dose was responsible for significantly higher serum levels of TNF*α*, IL-1, IL-6, IL-2, and MCP-1. Clearly, the mortality data paralleled the cytokine data indicating synergistic action of SEB and LPS, with LPS as the dominant factor in this mouse model of toxic shock.

 Cytokines are intracellular signaling proteins released from virtually all nucleated cells that regulate cell differentiation, proliferation, and inflammation [26]. Dysregulation of cytokine production has been associated with a variety of diseases, including autoimmune disorders, infectious diseases, cardiovascular diseases, asthma, and allergy. We examined the cytokines TNF*α*, IL-1, IL-6, IFN*γ*, and IL-2 as they are present in high levels in various animal models of shock [16–19]. The chemokines are chemoattractants produced by many cell types and are potent molecules involved in host defense as they direct leukocyte migration to sites of infection and injury [26]. We chose to examine two prototypical chemokines, MIP-2 and MCP-1, in this study as they influence leukocyte migration in various animal models of infectious diseases and inflammation [27]. 

 In this study, we showed that significantly different levels of TNF*α* (95-fold, *P* < .0001), IL-6 (32-fold, *P* < .0001), MIP-2 (15-fold, *P* < .0001), and MCP-1 (7-fold, *P* < .0001) were present in survivors and nonsurvivors among SEB, LPS, and SEB plus LPS treated animals early after toxin exposure. Most notably, a near 2 log higher level of serum TNF*α* was seen early in nonsurvivors. Significantly elevated IFN*γ* (18-fold, *P* < .0001) and IL-2 (31-fold, *P* < .0001) occurred later, 21 hours after toxin exposure, in nonsurvivors compared to survivors in all treatment groups. At this later time point, sustained elevated IL-6 (220-fold, *P* < .0001), MIP-2 (15-fold, *P* < .0001), and MCP-1 (107-fold, *P* < .0001) were also observed in nonsurvivors. The synergistic action of SEB and LPS promotes moderate level of TNF*α* early and prolongs the release of IL-6, IFN*γ*, IL-2, MIP-2, and MCP-1 in nonsurvivors. The disordered immune response based on the exaggerated release of a combination of proinflammatory cytokines and chemokines early (TNF*α*, IL-6, MIP-2, and MCP-1) at 8 hours, followed by significant increase of IFN*γ* and the substained high levels of IL-6, MIP-2, and MCP-1 later appear to be critical in influencing survival in shock.
